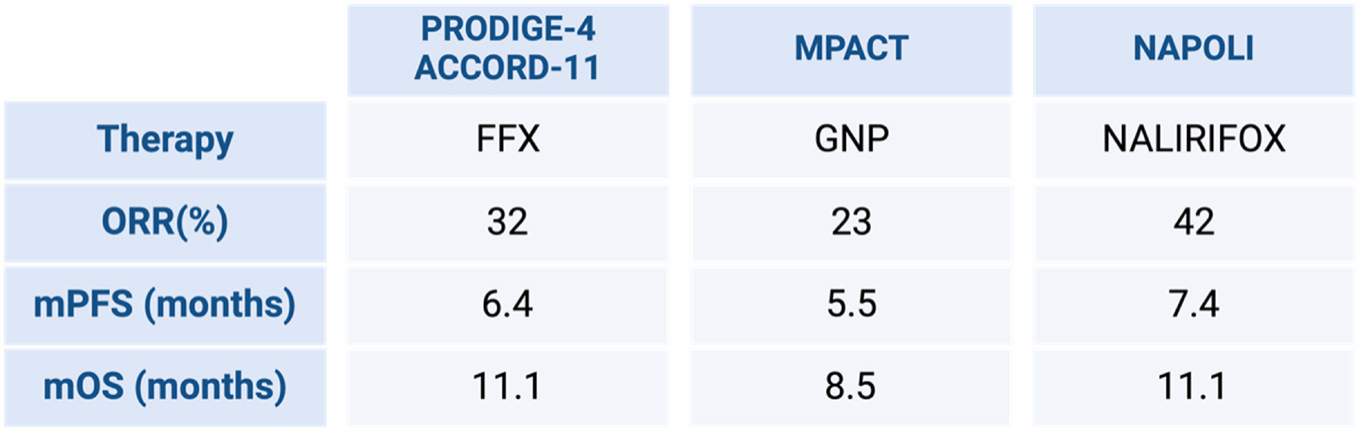# Pancreatic Cancer: Current Concepts, Trends, and Future Directions

**DOI:** 10.5152/tjg.2024.24544

**Published:** 2024-11-18

**Authors:** Patrick Wenzel, Carolin Mogler, Kıvanç Görgülü, Hana Algül

**Affiliations:** 1 Comprehensive Cancer Center Munich TUM, TUM University Hospital, Klinikum rechts der Isar, Technical University of Munich, Munich, Germany; 2 Internal Medicine II, TUM University Hospital, Klinikum rechts der Isar, Technical University of Munich, Munich, Germany; 3 Institute for Pathology, TUM University Hospital, Klinikum rechts der Isar, Technical University of Munich, Munich, Germany; 4 Institute for Tumor Metabolism, TUM University Hospital, Klinikum rechts der Isar, Technical University of Munich, Munich, Germany

**Keywords:** Pancreatic cancer, adjuvant therapy, neoadjuvant therapy, palliative therapy, genetic profiling, targeted therapy

## Abstract

Pancreatic ductal adenocarcinoma (PC) ranks among the deadliest cancers, with a less than 15% 5-year survival rate. Epidemiological studies project that it will become the second leading cause of cancer-associated mortalities in the following decades. The hallmarks of pancreatic cancer lead to tumor aggressiveness and therapeutic resistance. For this reason, the field has been focusing on multiple dimensions to generate better therapeutic approaches, including new adjuvant, neoadjuvant, and palliative concepts to extend the survival of PC patients. Over the last 2 decades, clinical trials have significantly improved disease prognosis and patient survival. To achieve better outcomes and to deeply understand the therapeutic approaches, molecular tumor boards have become crucial for deeper exploitation of tumor genetics and tumor biology, providing better stratification markers for therapeutic regimens. Using recently developed targeted therapies, such as KRAS inhibitors, the field has gathered momentum and been tooled up with the help of new sequencing technologies. Therefore, researchers and clinicians have geared up for the battle against PC. This review will systematically discuss recent developments in adjuvant, neoadjuvant, and palliative treatment modalities. Moreover, the paradigm-shifting importance of genetic profiling on pancreatic cancer. will be explained through a showcase to frame future directions.

**Main Points**

- Among the deadliest types of cancer, pancreatic cancer (PC) is seeing an increase in incidence.
- In most patient scenarios, pancreatic cancer is diagnosed in the metastatic stage, while only slightly more than a third of the patients display primary resectable or locally advanced scenarios.
- Neoadjuvant concepts in primary resectable and locally advanced scenarios are currently tested in clinical trials to improve outcomes in these patients.
- In the palliative setting, therapeutic options are limited, but recent developments have gained significant momentum regarding personalized approaches.
- Molecular tumor boards and genetic testing of tumor patients have, therefore, become a new and essential pillar in the clinical management of PC to define new stratification strategies for targeted therapies.

## Introduction

Adenocarcinoma of the pancreas, known as pancreatic ductal adenocarcinoma (PC), remains a tumor disease with a very poor prognosis. Almost every patient suffering from PC will die as a direct result of the tumor. Compared to other solid tumors, the 5-year survival rate has changed only marginally in recent decades and is below 15% across all stages. According to epidemiological projections, this number will remain at this level in the coming years. In addition, due to an increasing incidence, PC will rank second behind lung cancer concerning cancer-associated mortality by 2040.

**Figure 1.**
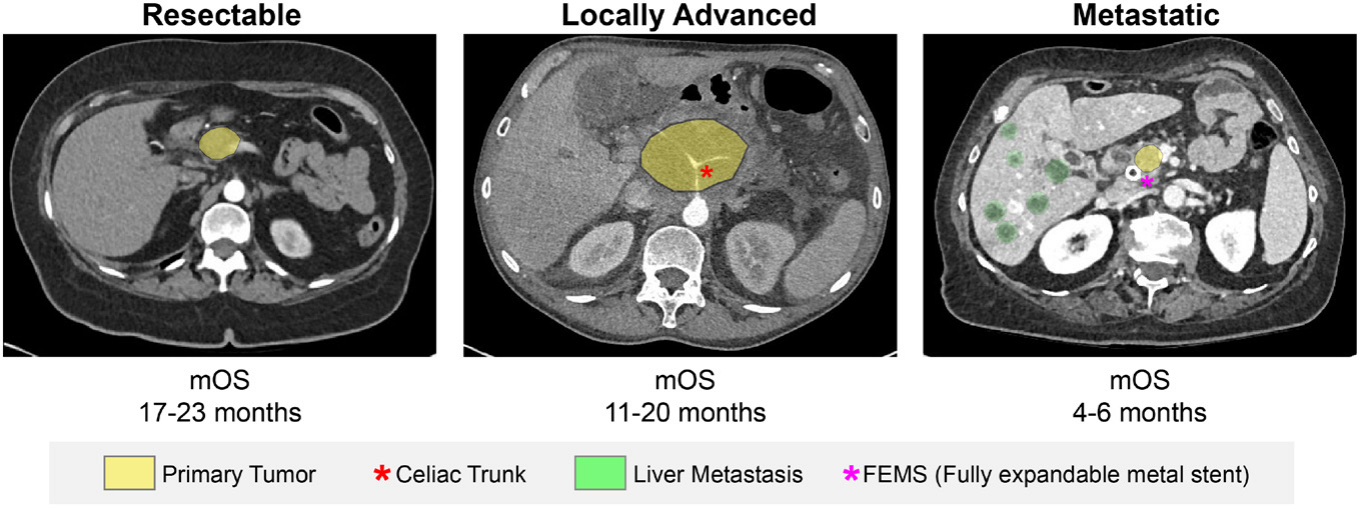
Clinical stages of pancreatic cancer, including resectable, locally advanced, and metastatic stages with overall survival in months (mOS).

Pancreatic ductal adenocarcinoma, as one of the most lethal cancer types, is diagnosed in 3 different scenarios: the primary resectable (PR), locally advanced, and metastatic settings. These scenarios vary not only in their prognostic outcomes but also in their therapeutic approaches (Figure 1).

**Figure 2.**
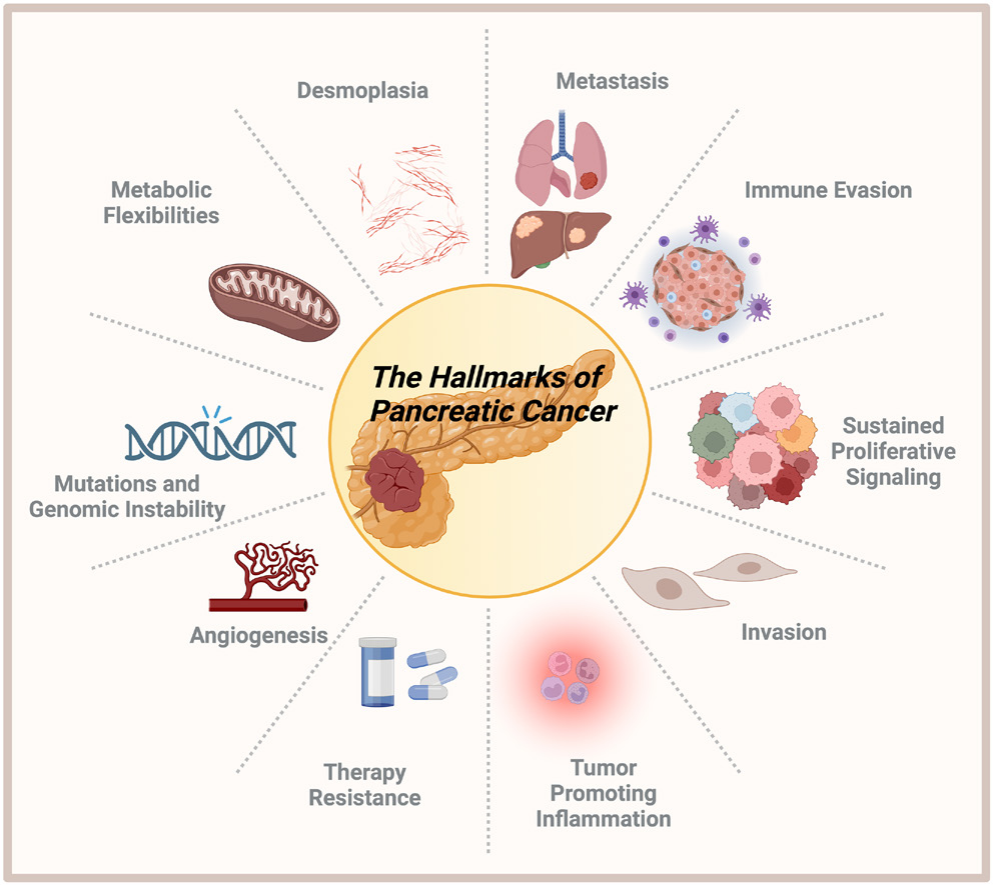
The hallmarks of pancreatic cancer comprise desmoplasia, metastasis, immune evasion, sustained proliferative signaling, invasion, tumor-promoting inflammation, therapy resistance, angiogenesis, mutations and genomic instability, and metabolic flexibility (created by BioRender).

Pancreatic ductal adenocarcinoma is notorious for its aggressiveness and bad prognosis. Pancreatic ductal adenocarcinoma is characterized by late diagnosis due to the lack of apparent symptoms and reliable early biomarkers. Genetic alterations, including KRAS, TP53, SMAD4, and CDKN2A, primarily drive the aggressiveness of the disease. Genetic background and dense desmoplastic stroma also create a hypoxic and immunosuppressive microenvironment that hinders drug delivery and immune cell infiltration.^1^ Moreover, PC displays considerable metabolic adaptability to its microenvironment, enabling it to endure in low-nutrient and oxygen-depleted conditions (Figure 2).

To survive these conditions, PC cells deregulate their cellular energetics and depict several metabolic flexibilities.^2^ For instance, switching between several metabolic cascades, such as oxidative phosphorylation, glycolysis, autophagy, and other scavenging pathways, including macropinocytosis, enables cancer cells to obtain essential nutrients for survival.^3^ During the interaction between tumor cells and their microenvironment, non-cell autonomous players such as fibroblasts, immune cells, pericytes, endothelial cells, islets, and other cell types increase the complexity of therapy response in pancreatic cancer. Cancer cells become more aggressive, invasive, and metastatic in their tumor-promoting inflammatory environment using immune evasion mechanisms.^4^ In the end, accumulating these hallmarks leads to therapy resistance and metastatic dissemination, shortening the survival of patients.

Current developments in therapeutic approaches continue to focus on chemotherapeutic agents in all 3 scenarios. This chapter presents an overview of these developments and opens the horizon for future concepts.

### Adjuvant Therapy

**Figure 3.**
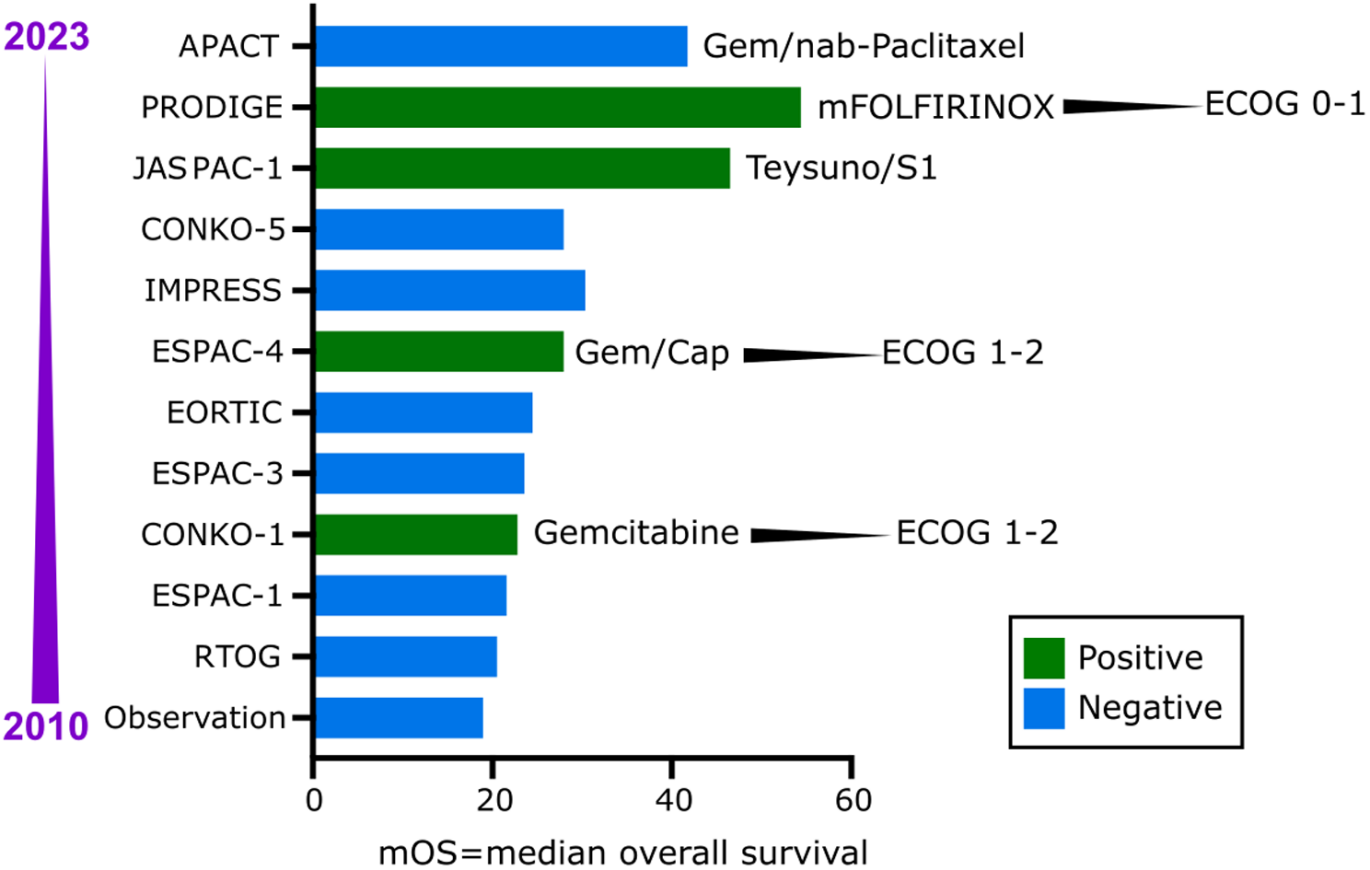
Clinical trials with positive and negative results depicting their median overall survivals during the last decades (between 2010 and 2023).

R1 resection and recurrence rates are high with an initial diagnosis of primary resectable pancreatic cancer (Figure 2). As a standard of care, adjuvant chemotherapy is carried out after successful resection to improve the outcome of these patient groups. In previous decades, various clinical trials have investigated the effectiveness of various therapies (Figure 3). Currently, the standard adjuvant treatment for patients in the good general condition is modified FOLFIRINOX (mFOLFIRINOX) (PRODIGE 24–ACCORD). At the same time, gemcitabine/capecitabine (ESPAC-4) or even gemcitabine monotherapy (CONKO-001) are further options for patients with a poorer ECOG.^5-7^

The phase 3, multi-institutional, randomized, open-label PRODIGE 24–ACCORD^8^ trial compared adjuvant mFOLFIRINOX (n = 247) with gemcitabine monotherapy (n = 246) in patients with resected PC.^5^ At a median follow-up of 33.6 months, the mFOLFIRINOX group exhibited significantly better disease-free survival (DFS) (median, 21.6 vs. 12.8 months; 3-year rate, 39.7% vs. 21.4%). After 3 years, the DFS rate was 39.7% in the mFOLFIRINOX group and 21.4% in the gemcitabine group. The mFOLFIRINOX group also showed better overall survival (median, 54.4 vs. 35.0 months; 3-year rates, 63.4% vs. 48.6%). Severe adverse effects (grade 3/4) were reported in 75.9% of mFOLFIRINOX-treated patients and 52.9% of gemcitabine-treated patients. The 5-year update confirmed the greater efficacy of the mFOLFIRINOX regimen over gemcitabine monotherapy.^9^ With a median follow-up of 69.7 months, the mFOLFIRINOX group showed significantly enhanced DFS (median, 21.4 vs. 12.8 months) and overall survival (median, 53.5 vs. 35.5 months). The mFOLFIRINOX group demonstrated markedly superior metastasis-free survival compared to the gemcitabine group (median, 29.4 vs. 17.7 months) and showed significantly improved cancer-specific survival (median, 54.7 vs. 36.3 months). The multicenter randomized trial ESPAC-4 assessed adjuvant gemcitabine plus capecitabine (n = 364) versus gemcitabine monotherapy (n = 366) in patients with resected PC.^6^ The gemcitabine and capecitabine group exhibited significantly better overall survival than the gemcitabine alone group (median, 28.0 vs. 25.5 months). The adjuvant gemcitabine and capecitabine group was found to have a 21% reduction in mortality following recurrence compared to the gemcitabine group.^10^

In the results of the CONKO-01 study, gemcitabine monotherapy is reserved solely for patients with impaired functional status. Gemcitabine demonstrated significantly better DFS than the observation group (median, 13.4 vs. 6.9 months). With a median follow-up of 136 months, adjuvant gemcitabine treatment showed improved overall survival (5-year overall survival rate, 20.7% vs. 10.4%; 10-year overall survival rate, 12.2% vs. 7.7%).^7^

The most recent study to evaluate a new regimen in the adjuvant setting was the Adjuvant nab-Paclitaxel Trial adjuvant pancreatic adenocarcinoma clinical trial (APACT). The APACT trial compared nab-paclitaxel plus gemcitabine versus gemcitabine alone in patients with resected PC. Nab-paclitaxel plus gemcitabine is a recognized therapy in the palliative setting and has proven efficacious in the Metastatic nab-Paclitaxel Trial (MPACT). The global study enrolled 866 treatment-naive patients who had undergone macroscopic complete resection. Patients were randomly assigned to receive, within 12 weeks of surgery, nab-paclitaxel at 125 mg/m^2^ plus gemcitabine at 1000 mg/m^2^ or gemcitabine alone at 1000 mg/m^2^ for 6 cycles. The primary endpoint was radiologically assessed DSF, evaluated independently without reviewers’ awareness of the clinical context. According to an independent evaluation, APACT failed in its principal aim of enhancing DSF. Median DFS was 19.4 vs. 18.8 months and 16.6 vs. 13.7 months, respectively. Independently assessed DFS was evaluated by a radiologist who was unaware of the treatment assignment. Conversely, treating physicians determined investigator-assessed recurrence using all available clinical data.^11^

Radiation’s role in adjuvant therapy remains a topic of debate. There is a lack of trials comparing a radiation and chemotherapy (CRT) approach to modern chemotherapy regimens. The ESPAC-1 trial found no survival change between 175 patients who received postoperative CRT and 178 who did not (median overall survival 15.5 vs. 16.1 months, respectively).^12^ Notably, in the subsequent intent-to-treat analysis of the 289 patients, there was a trend toward poorer survival for the group receiving CRT.^13^ A meta-analysis of 9 randomized trials comparing 6 different adjuvant strategies showed a lack of precision, making it hard to draw meaningful conclusions about the benefit of CRT.^12^

Thus, adjuvant chemotherapy trials have shifted their strategy from single-agent chemotherapy to more effective combination modalities.

### Neoadjuvant Therapy

Neoadjuvant concepts in solid gastrointestinal malignancies, such as colorectal or gastro-esophageal cancers, have been increasingly adopted in various settings and accepted as standards to improve the outcome after surgery. Therefore, in recent years, a shift has occurred from immediate surgery with adjuvant therapy to a neoadjuvant approach for patients with resectable (RPC) or borderline resectable PC (BRPC). Numerous studies have been unleashed to test the relevance of such concepts. Yet, comparing neoadjuvant and adjuvant trials is challenging because of a significant difference in patient selection.^14^ Neoadjuvant therapy at this stage remains of great interest for various reasons:

- Higher R0 resection rates after pretreatment.
- Better operability due to prior chemotherapy.
- Better patient compliance with chemotherapy compared to adjuvant therapy after major tumor surgery.
- Utilization of an early systemic effect and early response assessment.
- Avoidance of a stressful and unnecessary operation in those patients who progress very rapidly under chemotherapy.

Nevertheless, progression to an inoperable stage during neoadjuvant treatment poses a risk of losing the opportunity for curative surgery. While numerous trials have explored neoadjuvant therapy in RPC and BRPC, substantial variations in patient selection, study biases, different treatment regimens, and unequal comparisons make cross-study validations challenging.

The SWOG/NCI S1505 trial, which randomized patients with RPC to the neoadjuvant FOLFIRINOX group (n = 55) and gemcitabine+nab-paclitaxel group (n = 47), did not show better overall survival with chemotherapy prior to the operation, compared with the data from adjuvant treatment studies in RPC.^15^ Likewise, the PANACHE01-PRODIGE48 trial, which tested the neoadjuvant regimen of mFOLFIRINOX (n = 70) or FOLFOX (n = 50) relative to the current standard treatment (n = 26) in patients with RPC, failed to demonstrate a significant difference in 1-year survival rates (mFOLFIRINOX: 84.1%, FOLFOX: 71.8%, and upfront surgery: 80.8%).^16^ However, there was a 10% increase in the 1-year event-free survival rate (51.4% vs. 41.7%) and a 3-month improvement in event-free survival (median, 12.4 vs. 9.2 months) between the neoadjuvant mFOLFIRINOX group and the upfront surgery group. The German NEONAX study validated the outcome of patients with RPC receiving gemcitabine plus nab-paclitaxel, either perioperatively or adjuvant.^17^ The primary endpoint of the DFS rate (intention-to-treat population) of 55% at 18 months was not reached in both groups (33.3% vs. 41.4%).

Colleagues from the Netherlands published the first large, randomized phase III study on this topic. Two hundred forty-six patients were treated prospectively as part of the PREOPANC-1 study in the Netherlands.^18^ Patients were randomized between preoperative radiochemotherapy (RCTX) (“neoadjuvant”) and primary surgery, followed by adjuvant CTX with gemcitabine (“control”). The RCTX consisted of 3 cycles of gemcitabine, with the second cycle accompanied by radiotherapy (RTX) with 15 × 2.4 Gy to the primary tumor and suspicious lymph nodes. Although the primary study objective, namely a significant improvement in overall survival (OS) through neoadjuvant treatment in the overall population, was not achieved, all secondary endpoints were improved—with better overall compliance—so that the neoadjuvant treatment regimen appears superior to the primary surgery followed by adjuvant CTX. Chemotherapy with gemcitabine in the adjuvant setting no longer meets the standard, which is why the follow-up study PREOPANC-2 was launched and recently published with the initial results.^19^ This multicenter, randomized phase III study included patients with BRPC and RPC from 19 Dutch institutes. Patients received FOLFIRINOX followed by surgery without adjuvant therapy or 3 cycles of neoadjuvant gemcitabine with hypofractionated radiotherapy (36 Gy in 15 fractions during the second cycle), followed by surgery and 4 cycles of adjuvant gemcitabine. Three hundred seventy-five patients were included, of whom 188 were assigned to the FOLFIRINOX arm (n = 188) or the CRT arm (n = 187). After a median follow-up of 41.7 months with 254 events, the median OS was 21.9 months in the FOLFIRINOX arm and 21.3 months in the CRT arm (HR: 0.87; 95% CI: 0.68-1.12, *P* = .28). Resection rates were 77% in the FOLFIRINOX arm and 75% in the CRT arm (*P* = .69). Serious adverse events rates were 49% in the FOLFIRINOX arm and 43% in the CRT arm (*P* = .26). Thus, the study showed that neoadjuvant chemotherapy with FOLFIRINOX does not improve overall survival compared to neoadjuvant gemcitabine-based radiochemotherapy in patients with BRPC and RPC. The role of radiation seems to be even more complex. The Alliance A021501 validated the role of stereotactic body radiation (SBRT) in patients with BRPC.^20^ One group received 8 cycles of FOLIFRINOX, and the other received 7 cycles of FOLFIRINOX, followed by SBRT to a dose of 33-40 Gy in 5 fractions or 25 Gy in 5 fractions. The study was designed to independently assess the 18-month overall survival of each arm compared to a historical control of 50% (i.e., median survival of 18 months). The study was closed early due to futility analysis after only 10 of 30 patients in the FOLFIRINOX, followed by the SBRT group, underwent an R0 resection. At the final analysis, the 18-month overall survival was 66.7% in the FOLFIRINOX group and 47.3% in the FOLFIRINOX followed by the SBRT group. The rate of grade 3+ toxicity was 57% in the FOLFRINOX group vs. 64% in the combination cohort. It is unclear why patients who received radiation had worse outcomes compared to patients who received mFOLFIRINOX alone, although there are several concerns with the study.

The recently presented data from the NorPACT trial (randomized phase II trial with patients from 12 Scandinavian centers) also show no benefit of neoadjuvant chemotherapy with FOLFIRINOX over primary resection of localized tumors.^21^ Patients with resectable pancreatic head cancer were randomized to receive 4 cycles of neoadjuvant FOLFIRINOX followed by surgery and 8 adjuvant cycles of mFOLFIRINOX. In the other group, patients received primary surgery followed by 12 cycles of adjuvant mFOLFIRINOX. Overall survival at 18 months was the primary endpoint (intention-to-treat (ITT)). The median overall survival after ITT was 25.1 months (95% CI: 17.2-34.9) in the neoadjuvant arm and 38.5 months (95% CI: 27.6—not reached) in the primary surgical arm (*P* = .096). This study does not prove any benefit for neoadjuvant FOLFIRINOX in resectable pancreatic head cancer compared to prior surgery, either. The results of further ongoing trials (Table 1) in this field will hopefully help to shed a bright and clear light on this still controversial topic.

Thus, primary surgery followed by adjuvant therapy (preferably FOLFIRINOX) remains the standard for localized PC.

It is widely accepted that patients with locally advanced pancreatic cancer (LAPC) require neoadjuvant treatment. However, the optimal preoperative treatment for LAPC is unknown.^22^ The FOLFIRINOX regimen has become the preferred option as a neoadjuvant-intended protocol.^23^ Patients with LAPC benefit from resection after induction chemotherapy, even if complex vein resection/reconstruction is required. The type of pre-treatment is currently being investigated in ongoing clinical trials. Studies like NEOLAP or JCOG 1407 show the benefit of chemotherapy. In the NEOLAP trial, patients with LAPC received induction chemotherapy with either nab-paclitaxel plus gemcitabine or sequential FOLFIRINOX, followed by surgical exploration. About two-thirds of patients in both groups proceeded to surgical exploration. The complete macroscopic tumor resection rate was 35.9% in the nab-paclitaxel and gemcitabine group and 43.9% in the sequential FOLFIRINOX group.^14^ The JCOG 1407 was designed to compare the gemcitabine and nab-paclitaxel (GNP) regimen with FOLFIRINOX in LAPC.^24^ Both regimens achieved similar efficacy and showed better 1-year survival than gemcitabine monotherapy. GNPseemed to have a better disease control rate, CA19-9 response, and a more favorable profile of gastrointestinal toxicity.

The CONKO-007 trial aimed to illuminate the role of radiation in the neoadjuvant setting of LAPC patients.^25^ The trial revealed that adding radiotherapy after induction chemotherapy improves the R0 CRM—resection and pCR rate, albeit the R0 resection rate as the primary endpoint did not reveal significant differences. Interestingly, of the 525 patients, 190 patients were excluded due to progression or toxicity after the induction of chemotherapy. The CRT arm had significantly increased hematological toxicities and non-hematological toxicities were comparable. No significant differences in progression-free survival (PFS) or OS were observed between both cohorts.^26^

While pretreatment of LAPC is generally accepted and the decision to provide surgery in the first place is refuted, it remains to be seen which neoadjuvant treatment will evolve and set standards in future trials.

### Palliative Therapy

**Figure 4.**
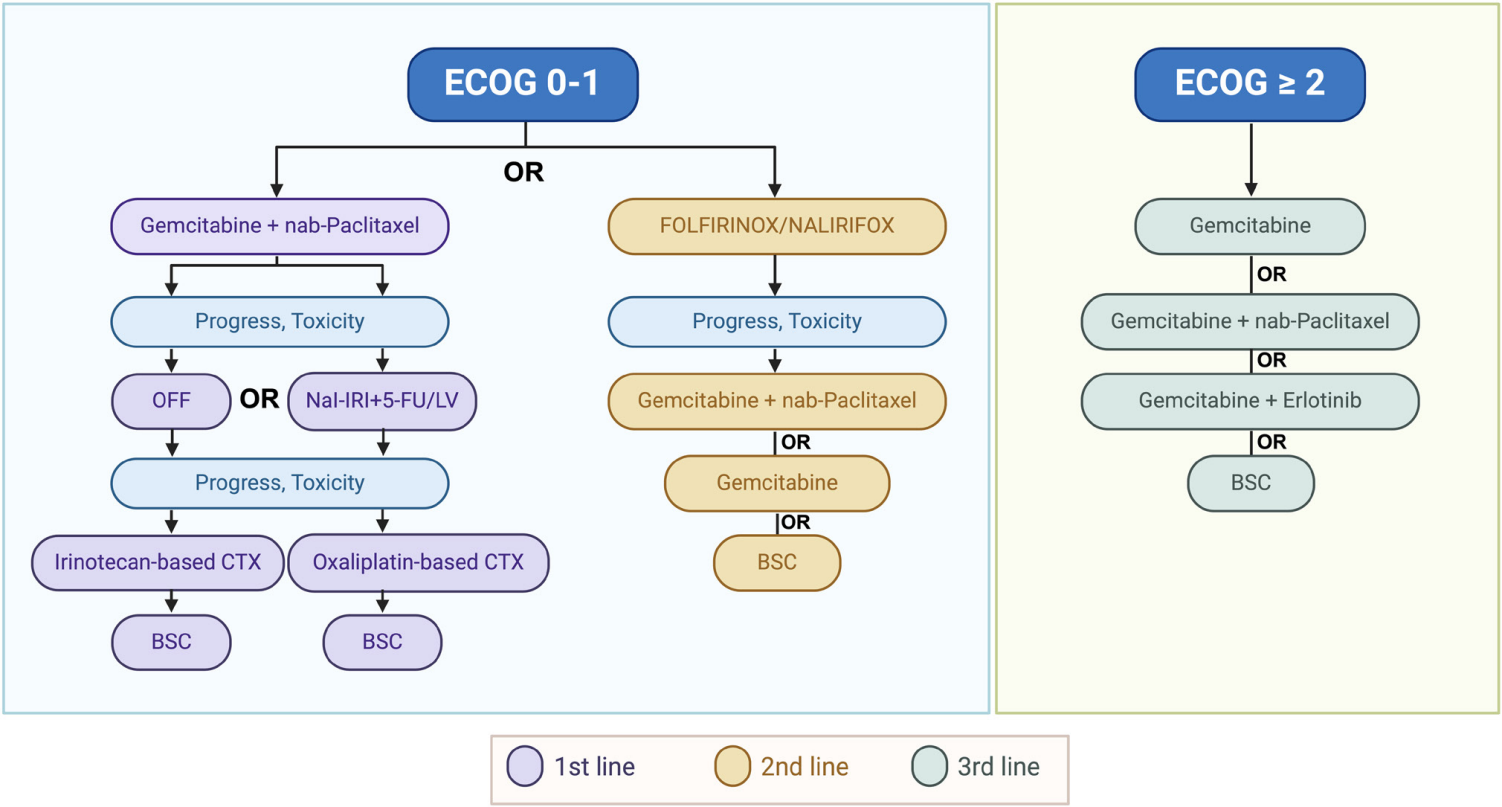
Therapeutic options in metastatic pancreatic cancer (created by BioRender).

For metastatic pancreatic cancer (mPC), polychemotherapies still remain the standard in the first line (Figure 4). In the phase III PRODIGE/ACCORD 11 clinical trial, FOLFIRINOX (FFX), demonstrated improved survival outcomes compared to gemcitabine monotreatment (Table 2). In the FFX arm, overall survival reached 11.1 months compared to 6.8 months in the control group. Thus, the median PFS was 6.4 months for the FOLFIRINOX group and 3.3 months for the gemcitabine group.^5^ Although quality of life (QoL) deteriorated in both treatment arms, patients who received FFX had better QoL than gemcitabine.^27^ Based on these results, FFX is recommended for metastatic PC in younger (less than 75 years) patients in good clinical conditions (ECOG PS 0-1). However, this chemotherapy regimen is associated with significant toxicities, notably grade 3 or 4 neutropenia in 45.7% of patients in the pivotal study. While the ACCORD11/PRODIGE4 trial reported a 5.4% rate of febrile neutropenia, real-world studies have found rates varying from 7 to 26%.^28-30^ The usage of G-CSF as primary prophylaxis in patients treated for mPC ranges between 4.9 and 100% of patients, according to various retrospective series.

Interestingly, many retrospective studies have evaluated mFFX as a first-line treatment widely used in clinical practice. In the mFFX protocol, bolus 5-FU is omitted, and the dose of irinotecan is reduced.^29,31^ Modified FOLFIRINOX has demonstrated reduced adverse events and similar efficacy to FFX. Thus, mFFX has been widely implemented into clinical practice for first-line palliative settings.^32^

Within the large scale of MPACT, it has been shown that GNP significantly improved both OS and PFS compared to gemcitabine alone (Table 2). Among 861 patients with metastatic PC, randomization was to GNP or gemcitabine monotherapy.^33^ The combination regimen resulted in better median OS (8.5 vs. 6.7 months; HR: 0.72), median PFS (5.5 vs. 3.7 months; HR: 0.69), and overall response rate (23% vs. 7%). Notably, grade 3 or 4 peripheral neuropathy and hematotoxicity were significantly higher with the GNP combination than with gemcitabine alone. Consequently, the U.S. Food and Drug Administration (FDA) approved the combination of nab-paclitaxel and gemcitabine as a first-line chemotherapy option for metastatic PC in 2013.

There are no prospective randomized trials directly comparing the 2 regimens (FFX vs. GNP), and analyses of non-randomized “real world” studies to date have not shown a significant advantage of one regimen over the other. Consequently, there is no clear preference between the two.^34^

The NAPOLI-3 study moves towards a direct comparison between the 2 protocols.^35^ In the FFX arm, the liposomal formulation known from the NAPOLI-1 study was used instead of the standard irinotecan (Table 2).^36,37^

The so-called NALIRIFOX was then compared with the GNP protocol. Overall survival in months (mOS) was 11.1 months by NALIRIFOX versus 9.2 months by nab-paclitaxel-gemcitabine. Grade 3 or worse treatment-related side effects were noted in 87% of the NALIRIFOX arm and 86% of the nab-paclitaxel/gemcitabine arm, with treatment-related deaths in 6 (2%) and 8 (2%) patients, respectively. Therefore, the frequency of toxicities was not different; only the spectrum seemed to vary. Regarding the primary endpoint of OS, the NAPOLI-3 study is positive. The survival advantage is statistically significant, although only moderately strong (11.1 vs. 9.2 months).^35^ However, the question of whether there are differences in effectiveness between NALIRIFOX and classical FFX remains open.

Interestingly, the Japanese study JCOG1611 (GENERATE) investigated the superiority of mFFX and S-IROX (S1, irinotecan, oxaliplatin) over GNP in metastatic or recurrent pancreatic cancer. in a phase II/III trial setting.^38^ The results showed an advantage of GNP over both regimens. The mOS was 17.1 months in the nab-paclitaxel plus gemcitabine arm, 14.0 months in the mFFX arm, and 13.6 months in the S-IROX arm. Due to the less tolerability of Asian patients towards mFFX, general conclusions cannot be drawn, and interpretation of these results is therefore limited. This is consistent with the detailed toxicity analyses. Both triplet therapies (mFFX, S-IROX) exhibited higher rates of the most common non-hematologic toxicities ≥ grade 3, such as anorexia, with incidences of 22.8% in the mFFX arm and 27.6% in the S-IROX arm. In comparison, these toxicities seem to occur significantly less frequently in the GNP arm (5.2%) than in the mFFX or S-IROX arm.

Over the last few years, the proportion of patients who physically qualify for second-line therapy due to their general condition has gradually increased. Around 50% of patients who have progressed during first-line therapy receive follow-up therapy. Second-line therapy choices depend on the initial treatment, but options are limited. Importantly, it has not been definitively established that subsequent chemotherapy improves survival after the failure of first-line chemotherapy.^39^ The only second-line chemotherapy that has been officially greenlit by the U.S. FDA was established in the NAPOLI-1 trial.^37^ Liposomal irinotecan combined with 5-fluorouracil/leucovorin (nal-IRI+5-FU/LV) has demonstrated an improvement in OS compared to 5-FU/LV alone (median OS of 6.1 months versus 4.2 months) in patients with metastatic pancreatic cancer who have not responded to gemcitabine-based chemotherapy.^36^ The combination of nal-IRI+5-FU/LV also showed advantages in median PFS, objective response rate, and disease control rate. The 1-year overall survival rates were approximately 26% for the nal-IRI+5-FU/LV group and 16% for the 5-FU/LV group. Factors associated with long-term survival in the nal-IRI+5-FU/LV cohort included a Karnofsky performance status of 90 or higher, age 65 or younger, lower CA19-9 levels, a neutrophil-to-lymphocyte ratio of 5 or less, and the absence of liver metastases.^36^

In the CONKO-003 trial, the study group assessed the effectiveness of second-line oxaliplatin combined with 5-FU and folinic acid (OFF regimen) versus FF alone.^40^ After progressing on first-line gemcitabine monotherapy, patients were randomly assigned in a 1 : 1 ratio to receive weekly infusional FF for 4 out of every 6 weeks (n = 84) or the same regimen supplemented with oxaliplatin 85 mg/m^2^ IV on weeks 1 and 3 (n = 76). Following a median follow-up of 54.1 months, the phase III trial reported a median survival of 5.1 months in the OFF group compared to 3.3 months in the FF group (*P* = .10). Importantly, the Canadian PANCREOX phase III trial demonstrated the effectiveness of oxaliplatin in this second-line context by comparing the combination following the biweekly modified (m)FOLFOX6 schedule (n = 54) with the biweekly infusional FF regimen as per the de Gramont schedule (n = 54).^41^ There was no enhancement in the primary endpoint of PFS, with a median of 3.1 months compared to 2.9 months. Conversely, median overall survival favored the infusional FF arm (6.1 months versus 9.9 months). Remarkably, the discontinuation rate due to toxicity without disease progression was substantially higher in the mFOLFOX6 arm (20% vs. 2%).^41^

The reasons for the contradictory results of PANCREOX and CONKO-003 are manifold. Both studies enrolled patients who had previously received gemcitabine therapy. Despite the planned 1 : 1 randomization, the CONKO-003 trial exhibited an unexplained imbalance in participant numbers between the arms. Notably, eligibility for CONKO-003 required patients to demonstrate disease progression while on gemcitabine therapy. In contrast, the PANCREOX trial permitted the inclusion of patients who experienced progression either during or after gemcitabine treatment. This difference in eligibility criteria may explain why OFF appeared to be better tolerated in the CONKO-003 trial compared to mFOLFOX6 in the PANCREOX trial. Additionally, equal proportions of patients in both CONKO-003 arms received subsequent therapy, whereas in the PANCREOX trial, patients in the mFOLFOX6 arm were less likely to receive further therapy than those in the control arm (25% vs. 7%).

First-line therapy standards have advanced beyond gemcitabine alone to include FOLFIRINOX or gemcitabine with nab-paclitaxel. These developments have challenged the optimal second-line strategy for patients. Thus, several alternatives may be explored for patients who have received gemcitabine plus nab-paclitaxel and maintain a good performance status. Further convincing data are needed to establish the optimal post-progression approach for patients with advanced pancreatic cancer.

### Genetic Testing in Metastatic Pancreatic Cancer

#### Genetic Profiles:

Pancreatic cancer is an oncogene-driven tumor disease.^42^ The classic molecular profile is formed by a pathogenic KRAS mutation (90% of patients, predominantly codon 12) along with inactivation of one or more classic tumor suppressors (CDKN1A 20%, CDKN2A 90%, SMAD4 60-90%, TP53 50-70%). While tumor suppressors are still considered to be undruggable, recent developments in the field of KRAS targeting have turned out to be quite promising. Even if the majority of patients with pancreatic cancer do not have any additional treatment alternatives resulting from molecular characterization of the tumor disease, a subgroup with the highest probability of therapy-relevant molecular changes can be identified. The focus here is on the identification of tumors with

- defects in DNA repair mechanisms (mostly in homologous recombination),
- KRASG12C mutations, and
- alternative driver genes in KRAS wild-type tumors.

The American Know Your Tumor data has already demonstrated the clinical effectiveness of extended molecular pathology diagnostics in pancreatic cancer. In a cohort of 677 patients, therapeutically relevant molecular alterations were detected in 189 (27%), of whom 25% of the patients received targeted therapy. Survival in these patients was extended (median survival 2.58 years) as compared to patients who did not receive targeted therapy (1.51 years) or in whom no druggable molecular alteration could be detected (1.32 years).^43^

### Molecular Pathology and Diagnostic Pitfalls

In addition to imaging modalities and elevated CA19-9 levels, endosonographic biopsies of the primary tumor are often conducted to confirm the diagnosis. If a classic morphology is present in the biopsy, including atypical cells of irregularly formed glands with desmoplastic stromal reaction (+/− inflammation) and/or the presence of intraepithelial neoplasia with dysplasia, the diagnosis can be made on a FOLFIRINOX&E basis solely. If additional testing is required, immunohistochemical stainings are performed, including 2 cytokines (CK7 and CK20) and CA19.9. Again, depending on the morphology, metastasis must be excluded, which is rare in the pancreas but occasionally occurs (e.g., clear cell renal carcinoma, malignant melanoma). Alternatively, if enough tumor tissue is available and morphology has an atypical phenotype (e.g., pleomorphic or giant cell-rich tumors), KRAS mutation analysis can be performed, as independently of their morphology, the vast majority of PC harbor KRAS mutations.

While the tumor biopsy itself is usually sufficient for diagnosis, its utility for conducting DNA or RNA-based wider panel analysis is hampered due to low tumor cell content or strong desmoplastic stromal reaction. For all those patients who have undergone surgical resection, the tumor specimens may be used for comprehensive genetic analyses in the setting of cancer relapses. Even after chemotherapies, no significant changes in currently druggable alterations discussed in this review are to be expected. Currently, tissue-independent diagnostic procedures are also available with liquid biopsy, but unlike tissue-based procedures, they are not expected to become firmly established in clinical practice within the next few years.

### KRAS as Key Oncogene

Mutated KRAS appears to be the most important oncogenic driver of cancer development in the pancreas and other solid tumors. For decades, this oncoprotein was considered inaccessible for therapeutic purposes. Only in recent years has it been possible to identify a so-called Switch II pocket (SIIP) in the protein, which enables effective pharmacological inhibition.^44^ The majority of KRAS mutations in ductal pancreatic cancer are found in codons 12, 13, and 61, with the specific KRAS G12D, G12V, and G12R variants accounting for the majority (approx. 80%), while the therapeutically relevant G12C variant is only detected in approx. 2% of pancreatic cancers. In the so-called CodeBreaK 100 trial, a maximum of previously treated patients with a KRASG12C mutation were subjected to monotherapy with the new KRASG12C inhibitors in this study with Sotorasib. Even in these patients, disease control was achieved in about one-third of the cases; the median duration of the response was almost 6 months.^45^ As promising as the results are, it has become clearer in recent months that monotherapy with the generation of KRAS inhibitors will not be sufficient, and secondary resistance will be a problem.^46^ Therefore, current studies focus on novel panKRAS inhibitors or even degraders combined with various substances to inhibit so-called “downstream” effectors, as well as relevant receptor tyrosine kinases. For example, the new group of allosteric SHP2 inhibitors appears to be important in blocking the mutated KRAS-driven signaling pathway.^47^

### Non-Mutated Wild-Type KRAS

In approximately 10% of tumors, no KRAS mutation can be detected, with a mild dependence on the age of onset. In about 40%-50% of these cases, however, an alternative, potentially therapeutically useful, oncogenic alteration is found.^47^

**Figure 5.**
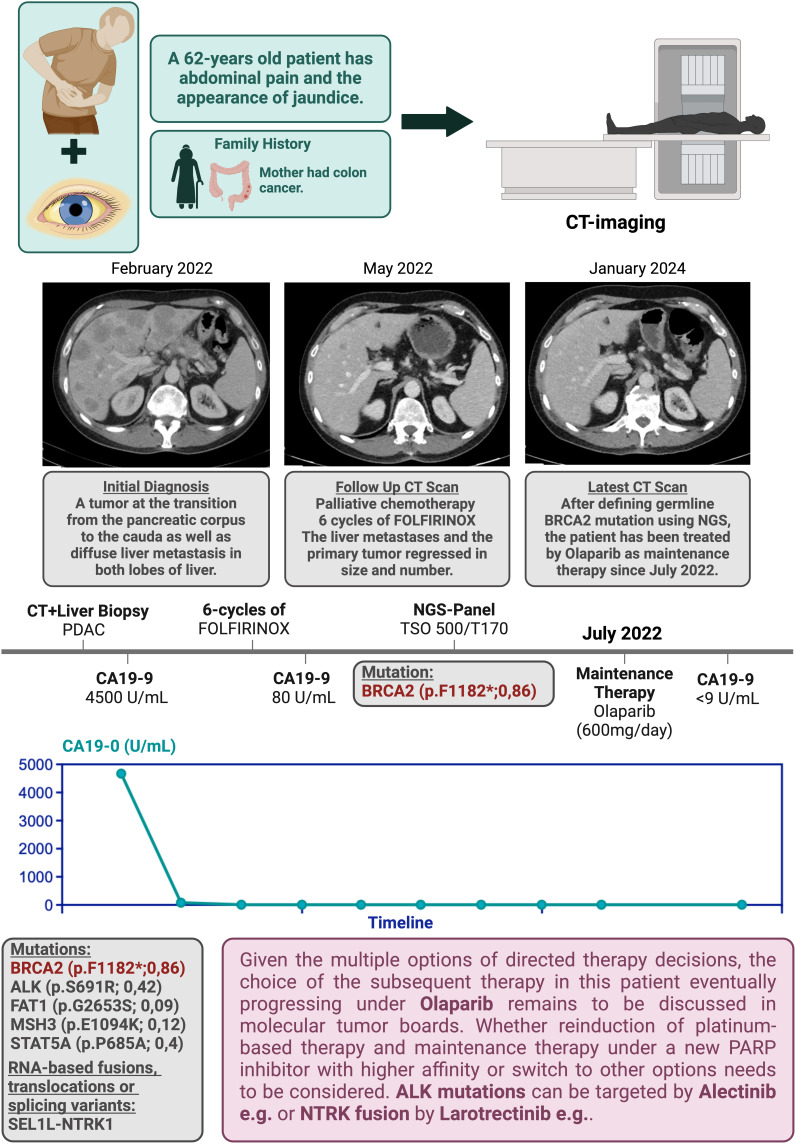
Identification of targetable genetic alterations in a PC patient following palliative chemotherapy with FFX. The total cycle number was 7. The last cycle has not been depicted in the sketch (created by BioRender).

The most frequent and actionable mutation was related to BRCA alterations that occur in wild-type and mutated KRAS tumors. The relevance of this genetic alteration was shown in the POLO trial.^48,49^ In the POLO trial, the BRCA gene germline mutations were utilized as a stratification marker to select a patient population that would benefit from targeted therapy with Olaparib based on the underlying DNA repair mechanism. Several studies confirmed the relevance of somatic mutations of BRCA and other DDR-associated genes.^50^ However, genetic analysis is more likely to unveil druggable mutations in wild-type KRAS tumors. The following case demonstrates the spectrum of multiple targetable genetic alterations (Figure 5).

The largest subgroups are BRAF alterations in which the classic BRAFV600E variant, the in-frame deletion variant BRAFN489_490del, SND1-BRAF fusions, and other rarer BRAF point mutations occur in approximately equal proportions. In addition to using classic BRAF and MEK inhibitors, non-specific inhibitors such as sorafenib can also be considered.

In addition to BRAF alterations, the spectrum of other driver mutations in KRAS wild-type pancreatic carcinomas is very broad and includes EGFR (mutations), ERBB2, and MET (amplifications), as well as various gene fusions in, e.g., FGFR1-4, NTRK1-3, ALK, ROS1, NRG1 with individual frequencies in unselected cohorts of well below 1%. Even though specific inhibitors are available, some of which have cross-entity approval (e.g., for NTRK fusions), PFS rates are only modest. Yet, given the aggressiveness of the tumor disease, this might be relevant for a subgroup of patients.

Due to the large number of expected alterations and the rarity of entity-specific variants, patients with KRAS wild-type diseases should be presented at a center experienced in molecular diagnostics and therapy.

The approach to treating pancreatic cancer depends on the tumor’s resectability. According to the current status, primarily resectable pancreatic carcinomas are primarily operated on, followed by adjuvant chemotherapy, preferably with FFX. Neoadjuvant therapy approaches should only be carried out within clinical trials. This does not apply to locally advanced pancreatic carcinomas. Here, primary resection is obsolete, and pre-treatment is mandatory. However, the type of pre-treatment is still unclear. Sequential therapy is now well-established for metastatic pancreatic carcinoma. Targeted therapies are limited, but future developments may change this area. Whether vaccination strategies based on mRNA technology or bacterial delivery will lead to further progress remains to be seen.

## Availability of Data and Materials:

The data that support the findings of this study are available on request from the corresponding author.

## Figures and Tables

**Table 1.** Clinical Trials (IDs are curated from ClinicalTrial.gov) with their Interventions, Comparators, and Patient Size (created by BioRender).

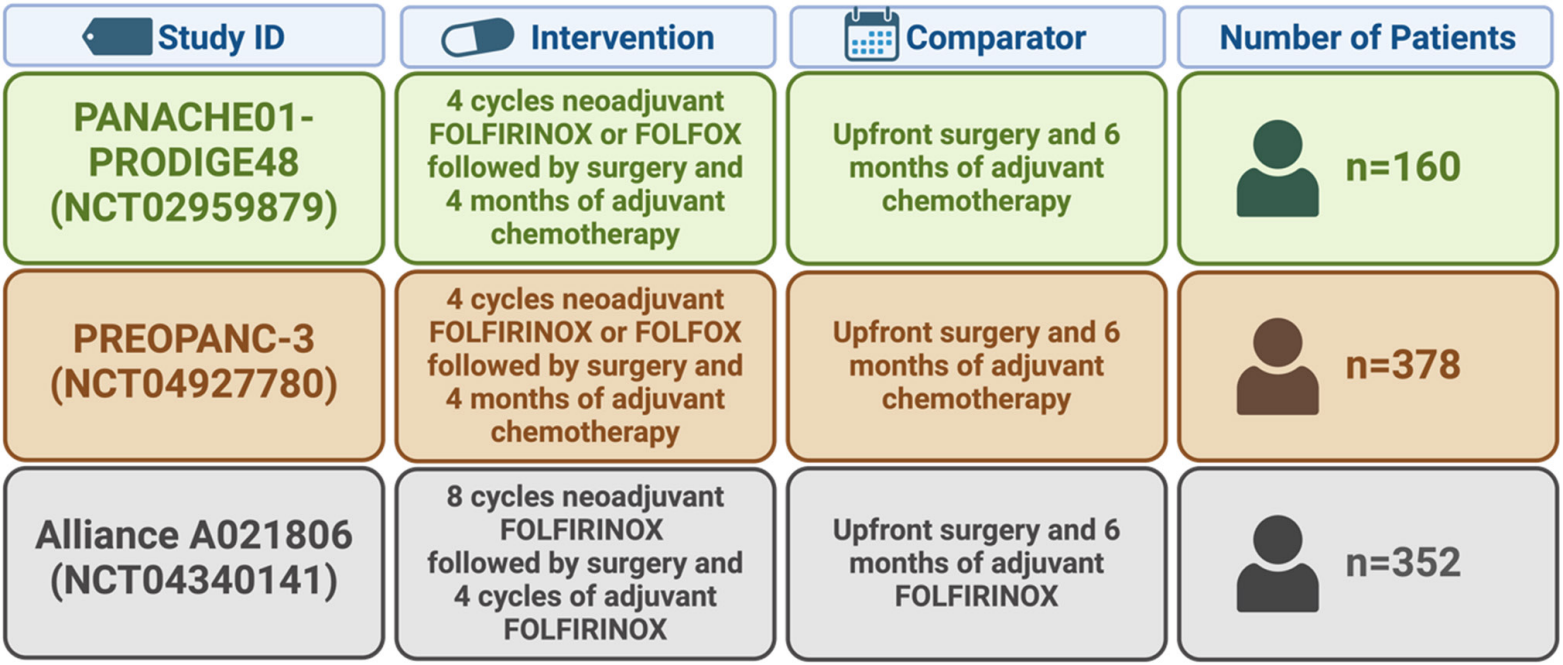

**Table 2.** Recent Clinical Trials with their Therapeutic Regimens and ORR (%), mPFS, and mOS (Created by BioRender). ORR, Overall Response Rate; mPFS, Progression-free Survival; mOS, Overall Survival in Months.